# Leveraging biomimetic synthesis strategy for next-generation dendritic cell nanovaccines

**DOI:** 10.20517/evcna.2022.35

**Published:** 2022-10-08

**Authors:** Yutian Xia, Jianzhong Zhang

**Affiliations:** State Key Laboratory of Molecular Vaccinology and Molecular Diagnostics, Center for Molecular Imaging and Translational Medicine, School of Public Health, Xiamen University, Xiamen 361102, Fujian, China.

**Keywords:** Cancer immunotherapy, extracellular vesicles, bioengineering, nanovaccine

## Abstract

The activation of CD8^+^ cytotoxic T-lymphocytes (CTLs) plays the central role in cancer immunotherapy, which depends on the efficient recognition of peptide-major histocompatibility complex (pMHC) by the T cell receptor (TCR) for the first signal, and B7-CD28 co-stimulating for the second signal. To achieve the potent immune stimulatory effect, a genetically engineered cellular membrane nanovesicles platform that integrates antigen self-presentation and immunosuppression reversal (ASPIRE) for cancer immunotherapy was designed. In preclinical mouse models, ASPIRE could markedly improve antigen delivery to lymphoid organs and generate broad-spectrum T-cell responses that eliminate established tumors. This review highlights that the ASPIRE system represents a novel strategy for personalized cancer immunotherapy.

Cancer immunotherapies, including immune checkpoint blockade (ICB), adoptive cell therapy (ACT), and cancer vaccines, have become powerful clinical options for cancer treatment^[1]^. Among these, cancer vaccines utilize tumor antigens to induce specific antitumor responses in the body through active immunization, stimulating immune protection mechanisms, and achieving the effect of treating tumors or preventing recurrence, which advances cancer treatment remarkably in recent years. Antitumor immune responses of cancer vaccines are based on the recognition of antigens by professional antigen-presenting cells (APCs) and the activation or initiation of naive antigen-specific T cells, especially CD8^+^ cytotoxic T lymphocytes (CTLs)^[2]^. Conventional tumor vaccines rely on random encounters between antigenic epitopes and host APCs, while inappropriate encounters might lead to immune response silencing^[3]^. In addition, the delivery of tumor antigens cannot maximize immunogenicity due to the complex and inefficient process of cross-presentation, which extremely limits the activation of CD8^+^ T cells. Although dendritic cell (DC) -based cancer vaccines can provide potent immunostimulatory activity, only a small fraction of activated DCs could migrate to draining lymph nodes (LNs) after injection; this restricts the application of cell-based cancer vaccines^[4]^. Furthermore, immune escape caused by tumor evolution, such as immune checkpoints, impairs the functions of antigen-specific CTLs in the fight against tumors^[5]^. Therefore, novel strategies are urged to further develop therapeutic cancer vaccines to overcome these challenges and rejuvenate the field of cancer immunology.

Upon harnessing the biomimetic synthesis strategies, protein cargos could be displayed on the cell surface^[6,7]^. Liu *et al*. demonstrated a novel nanovaccine platform derived from genetically engineered DCs cytomembrane nanovesicles (DCNVs) integrating antigen self-presentation and immunosuppression reversal (ASPIRE) to induce potent antitumor immunity^[8]^. As natural cell membrane-derived vesicles, DCNVs inherit unique biological functions of DCs, due to the presence of membrane-anchored proteins and immunological accessories. Whereas the excellent immune-stimulatory capacity in preclinical mouse models, we highlight that ASPIRE broads the landscape for the next-generation dendritic cell nanovaccines.

As a proof of concept, immature DCs were transducted with recombinant adenoviral vectors expressing chicken ovalbumin (OVA) for endogenous antigen loading by major histocompatibility class-I (MHC-I), during which DCs were activated. Following multistep density gradient ultracentrifugation, DCNVs were obtained. Mass spectrometry analysis revealed that the expression levels of a large number of co-stimulatory molecules (e.g., CD80, CD86, CD40) and chemokines (e.g., CCR2, CCR5, CCR7) were up-regulated in DCNVs, which contributed to the migration of DCNVs to LNs. By co-culturing DCNVs and CD8^+^ T cells, the authors found that DCNVs possess the complete surface functional proteins of mature DCs and could directly present antigens to naive T cells *in vitro*. Benefiting from the nanoscale size and expressed lymphatic homing molecules, DCNVs could be efficiently enriched in LNs, which facilitated adequate contact with T cells. Furthermore, the authors observed that DCNVs significantly elicited robust antigen-specific CTLs expansion and cytokine expression and effectively inhibited the growth of OVA-expressing tumors. It is worth noting that DCNVs also showed strong antitumor ability in mice with incapacitated antigen presentation, which indicated that DCNVs could present antigen directly to T cells without the participation of endogenous APCs.

To explore the utility of the DCNVs platform for neoantigen vaccination, the authors employed DCNVs transduced with three melanoma antigens to elicit broad-spectrum T-cell immune responses. Since such tumors tend to be highly aggressive and low immunogenic, tumor rejection cannot be observed despite DCNVs vaccination showing a significant delay in tumor growth. Further studies showed that this difference was mediated by an immunosuppressive tumor microenvironment in which both CD8^+^ T cells and tumor cells had high expression of PD-1 and its ligand PD-L1 in the draining LNs of tumor-bearing mice. In order to activate the immune response while reversing PD-1/PD-L1-mediated immunosuppression, the authors pre-expressed single-chain antibody fragment (scFv) against PD-1 on the surface of DCs and transferred three melanoma antigens, and the obtained DCNVs were called ASPIRE [Figure 1A]. ASPIRE treatment resulted in complete tumor regression in mice, while the tumor regression rate of DCNVs combined with the same amount of free PD-1 treatment was only about 40%. Interestingly, when B16F10 cells were injected into the tail vein of mice two months after the third vaccination, no tumor growth was observed in the ASPIRE-treated group, suggesting that ASPIRE induced a strong immune memory. CD28 is a key target of PD-1 blockade, and the co-stimulatory signal B7 molecule binds to the co-activating receptor CD28 on the surface of T cells to regulate T cell activation^[9]^. The authors used a Lewis lung cancer model sensitive to anti-PD1 therapy to study the important role of B7 molecules in the antitumor process of αPD1-DCNVs. B7 knockout αPD1-DCNVs did not inhibit tumor growth, and there was no significant difference in tumor progression and mouse survival compared with the free anti-PD1 treated group. This result suggested that B7 molecular co-stimulation is particularly important for enhancing anti-PD1 therapy of αPD1-DCNVs. Interestingly, the authors observed that ASPIRE could significantly decrease PD-1^+^CD38^high ^dysfunctional CTLs in tumor-bearing mice. They found that the responses of infiltrating T cells in the tumor immune microenvironment to anti-PD1 antibody and antigen stimulation differed in spatiotemporal order, and that ASPIRE could reverse immunosuppression [Figure 1B]. 

**Figure 1:**
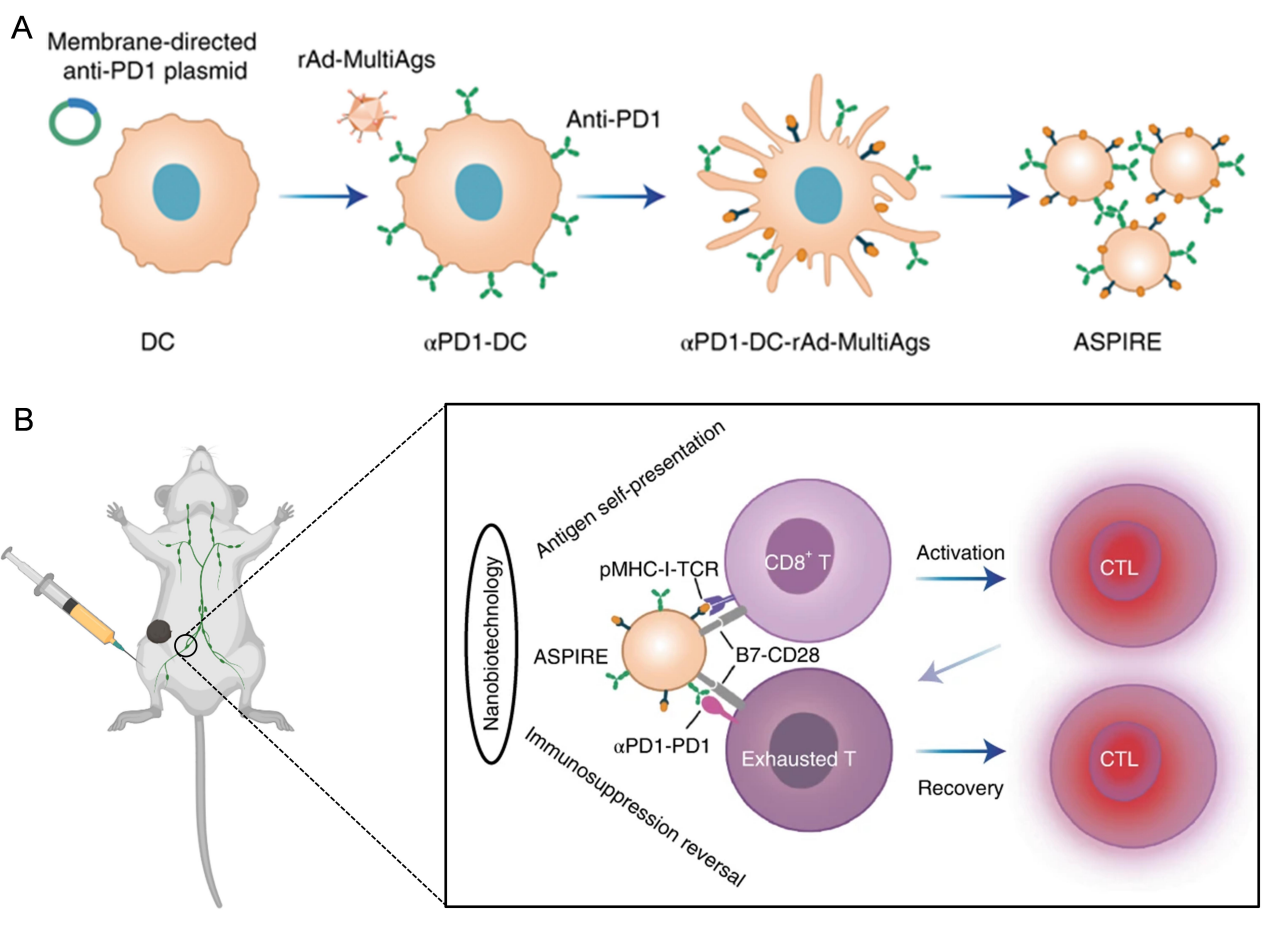
(A) Illustration of the process for preparing ASPIRE from DCs. DCNVs have nanoscale size, good stability, and a homing effect, thus enabling ASPIRE to be rapidly enriched in the lymphatic system. (B) Schematic describing ASPIRE for antigen self-presentation and immunosuppression blockade. ASPIRE: Antigen self-presentation and immunosuppression reversal; DCs: dendritic cells. Reproduced with permission. Copyright 2022, Springer Nature.

The potent tumor treatment effect elicited by ASPIRE in preclinical study promises an enhanced cancer immunotherapy strategy. To encourage the clinical translational, there are a few issues that still need to be considered. For dense solid tumors, it is difficult for circulating CTLs to be transported into the tumor microenvironment^[2]^, and without other immunotherapeutic interventions supporting T cell infiltration, DCNVs might be difficult to achieve the desired therapeutic effect. Although the development and innovation of sequencing technology and neoepitope prediction technology are beneficial to the clinical development of DCNVs, the heterogeneity and mutation of the tumor cells might increase the complexity of vaccine design^[10]^. At the same time, ASPIRE relies on relatively expensive sequencing and multiple transfections of DCs, so how to achieve cost-effectiveness and reduce the turnaround time (TAT) of patients is also worth consideration. More importantly, exploring the development of human allogeneic DCs-derived ASPIRE will be a more economical and feasible strategy to alleviate the suffering of patients and standardize the production on a large scale. Furthermore, some paralleled strategies, including bacteria-derived outer-membrane vaccines (OMVs) and whole-cell components nanovaccines, *etc.*, are other potential candidates for cancer immunotherapy^[11,12]^. We postulate that combining ASPIRE with OMVs may provide a rational combination for enhanced immunostimulatory and antitumor effects, whereas the large amounts of pathogen-associated molecular patterns (PAMPs) are anchored on OMVs.

In summary, this study revealed the mechanism by which co-stimulatory signaling reverses immune suppression and first described a novel nanovaccine formulation to activate the immune response and break immune tolerance simultaneously, which provides a new paradigm for personalized cancer immunotherapy.

## DECLARATIONS

### Copyright

© The Author(s) 2022.
